# Dietary total fat, fatty acids intake, and risk of cardiovascular disease: a dose-response meta-analysis of cohort studies

**DOI:** 10.1186/s12944-019-1035-2

**Published:** 2019-04-06

**Authors:** Yongjian Zhu, Yacong Bo, Yanhua Liu

**Affiliations:** 1 Department of Cardiology, The first affiliated hospital of Zhengzhou University, Zhengzhou, China; 2 Department of Nutrition, The first affiliated hospital of Zhengzhou University, No. 1 Eastern Jianshe road, Zhengzhou, 450052 Henan China; 3 Jockey Club School of Public Health and Primary Care, the Chinese University of Hong Kong, Hong Kong, China

**Keywords:** Fat, Fatty acids, Cardiovascular disease, Meta-analysis, Dose-response

## Abstract

**Background:**

Several epidemiological studies have investigated the association between dietary fat intake and cardiovascular disease. However, dietary recommendations based on systematic review and meta-analysis might be more credible.

**Methods and results:**

Pubmed, Embase and Cochrane library were searched up to July 1st 2018 for cohort studies reporting associations of dietary fat intake and risk of CVDs. By comparing the highest vs. the lowest categories of fat or fatty acids intake, we found that higher dietary trans fatty acids (TFA) intake was associated with increased risk of CVDs [RR:1.14(1.08–1.21)]. However, no association was observed between total fat, monounsaturated fatty acids (MUFA), saturated fatty acids (SFA), and polyunsaturated fatty acids (PUFA), and risk of CVDs. Subgroup analysis found a cardio-protective effect of PUFA in the studies that has been followed up more than 10 years [0.95(0.91–0.99), *I*^*2*^ = 62.4%]. Dose-response analysis suggested that the risk of CVDs increased 16% [1.16 (1.07–1.25), P_linearity_ = 0.033] for an increment of 2% energy/day of TFA intake.

**Conclusions:**

This current meta-analysis of cohort studies suggested that total fat, SFA, MUFA, and PUFA intake were not associated with the risk of cardiovascular disease. However, we found that higher TFA intake is associated with greater risk of CVDs in a dose-response fashion. Furthermore, the subgroup analysis found a cardio-protective effect of PUFA in studies followed up for more than 10 years.

**Electronic supplementary material:**

The online version of this article (10.1186/s12944-019-1035-2) contains supplementary material, which is available to authorized users.

## Backgrounds

CVDs, caused by disorders of the heart and blood vessels, are the leading cause of death globally [1]. It is estimated that 17.7 million people, which represented 31% of all global deaths, died from CVDs in 2015 [1]. Unhealthy lifestyles, especially dietary habits, are believed to play an important role in the development of heart disease [2–5]. National health institutions has traditionally recommended to reduce the intake of dietary fat to prevent CVDs [6]. In addition, the intake of trans-fat has been demonstrated to be associated with health outcomes, and it is recommended to reduce its consumption a minimum [7].

The mechanisms underlying the effects of dietary fat on CVD remain uncertain. Dietary fat might contribute to CVD via inflammatory and oxidative stress mechanisms. Polyunsaturated fatty acid (PUFA) can decrease the production of inflammatory and reactive oxygen species [8], saturated fatty acid (SFA) and trans fatty acids (TFA) can increase pro-inflammatory and oxidative stress [9, 10], excessive oxidative stress and inflammation contributes to the development of CVD [11, 12]. Several previous studies have investigated the relationship between dietary fat and cardiovascular risk [13–16], the PREvención con DIeta MEDiterránea study (PREDIMED) demonstrated that Mediterranean diets (MedDiets), which were high in polyunsaturated fatty acid (PUFA) and monounsaturated fatty acid (MUFA), low in saturated fatty acid (SFA) and trans fatty acids (TFA), were associated with reduced CVDs events compared with a low-fat control diet [13]. The Japan Collaborative Cohort Study for Evaluation of Cancer Risk (JACC) Study showed that SFA intake was inversely associated with mortality from stroke [17]. However, the Lipid Research Clinics Prevalence Follow-up Study suggested that dietary MUFA and PUFA intake were associated with reduced risk of CVDs mortality and morbidity, whereas dietary intake of trans-fat and SFA were associated with increased risk of CVDs [14]. The Prospective Urban Rural Epidemiological (PURE) study found that total fat and types of fat were not associated with cardiovascular disease, or mortality, whereas saturated fat had an inverse association with stroke. Global dietary guidelines should be reconsidered in light of these findings [18]. However, in the development of guideline, recommendations should be informed by systematic review and meta-analysis of all evidences and/or large randomized clinical trials. [19].

Thus, it is warranted to conduct a meta-analysis to investigate the effect of dietary fat intake on CVDs risk. In the current study, we summarized the evidence from cohort studies of the relation of dietary fat intake and CVDs risk, and evaluated the dose-response relationship of CVDs and dietary fat intake.

## Methods

### Search strategy

Two members of the research team (Yonjian Zhu and Yanhua Liu) systematically searched Pubmed, Embase and Cochrane library up to July 1st 2018. The following keywords were adopted: (dietary fat OR fat OR fatty OR monounsaturated fatty acid OR polyunsaturated fatty acid OR saturated fatty acid OR trans fatty acid) AND (Cardiovascular disease OR vascular disease OR ischemic heart disease OR coronary heart disease OR Miocardial infarction OR Arrhythmia OR atrial fibrillation OR stroke OR cerebral infarction OR cerebral hemorrhage OR Heart failure OR Hypertension OR Cardiomyopathy).

### Study selection

Two investigators (Yongjian Zhu and Yanhua Liu) independently reviewed all identified studies, and studies were included if they met the following criteria: (i) based on a cohort or nested case-control study design; (ii) the exposure of interest were dietary total fat or major fat subclasses (SFA, TFA, MUFA, or PUFA) intake; (iii) the outcomes of interest were CVDs and (iv) relative risk (RR) or hazard ratio (HR) with 95% confidence interval (CI) were reported (we present pooled results with HR). If data were duplicated in more than one study, the one with the most recent or with the most number of cases was included. Conflicts were resolved via a third author (Yacong Bo).

### Data extraction

Two authors (Yanhua Liu and Yacong Bo) independently extracted the following information: the surname of first author, year of publication, cohort name, country, method of assessing dietary fat intake, number of participation, association between dietary fat intake and outcome (HR or RR and 95% *CI*); the definition of outcome; and potential confounding variables adjusted for HR (RR) with 95% *CI* across increasing fat intake categories were extracted preferentially or, where these data were not available, risk estimates for continuous (linear) exposures were extracted. Conflicts were resolved via a third author (Yongjian Zhu).

### Statistical methods

For the highest vs. the lowest dietary fat intake and CVDs risk, we used the DerSimonian and Laird random effects model to pool RRs and its 95% CIs from the adjusted HRs or RRs. Subgroup and meta-regression analysis were also performed based on geographical location (Americas, Europe, or other); follow-up duration (< 10 years or ≥ 10 years); and whether the results were adjusted for age, sex, physical activity, energy intake, alcohol, smoking, or body mass index.

Potential publication bias was assessed by Egger’s test (*P* < 0.05) [20, 21]. The sensitivity analysis was performed by excluding 1 study at a time to evaluate whether the results could have been affected markedly by a single study.

For the dose-response association of dietary fat intake and incident cardiovascular disease, the method of restricted cubic splines with three knots at percentiles 25, 50, and 75% of the distribution was adopted [22]. The studies included in doses-response analysis should report the number of cases and person-years (or controls), and the RR (or HR) with the variance estimates for at least three quantitative exposure categories. If the distribution of cases or person years was not reported, we estimated them based on the quintiles definitions [23]. Moreover, we assigned the median or mean fat intake for each category to each corresponding RRs (or HRs) with 95% CI. When means and medians were not reported, the category midpoint would be used. When the lowest category was open-ended, we set zero to the lowest boundary, and when the highest category was open-ended, we set the same width of the adjacent category to it [23].

All analyses were conducted using STATA software (version 12.0; StatCorp, College Station, TX, USA) and a value of *P* < 0.05 was considered as statistically significant.

### Patient and public involvement statement

Patients and the public sector were not directly involved in this study.

## Results

### Literature search and study characteristics

**Fig. 1:**
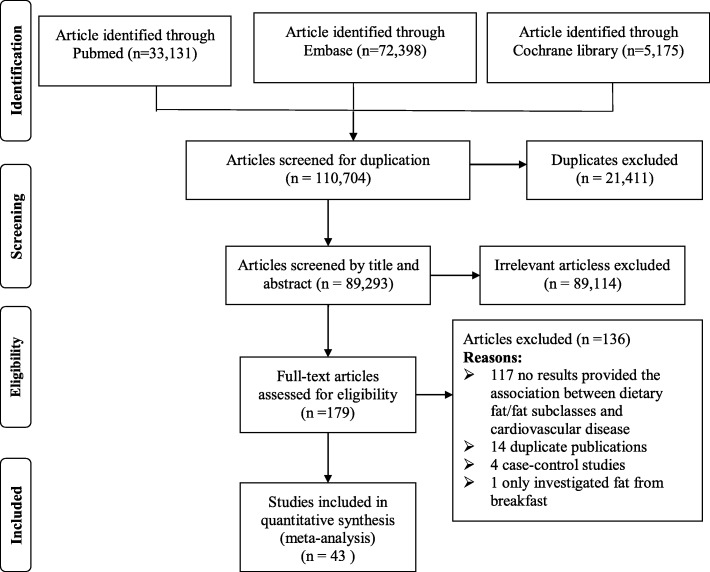
Search, screening and selection process of prospective cohort studies of dietary total fat and major fat subclasses and risk of cardiovascular disease

The detailed procedures of the article search and screening were presented in Fig. 1. Briefly, the search strategy retrieved 110,704 papers: 33,131 from Pubmed, 72,398 from Embase and 5175 from Cochrane library. After removing 21,411 duplicate articles, 89,293 articles were assessed. Among them, 89,114 articles were excluded after reviewing abstracts and titles, leaving 179 articles for full-text review. Of these, 136 papers were excluded due to: no results provided the association between dietary fat/fat subclasses and cardiovascular disease, duplicate publications, case-control studies, and only investigated fat from breakfast. In total, 43 publications [14, 15, 17, 18, 24–62] (63 studies, since 19 studies reported results for more than one study) that presented risk estimates for dietary total fat or major fat subclasses intake and CVDs risk were identified. The main characters of these studies are presented in Additional file 1: Table S1.

### Total fat intake and risk of CVDs

**Fig. 2:**
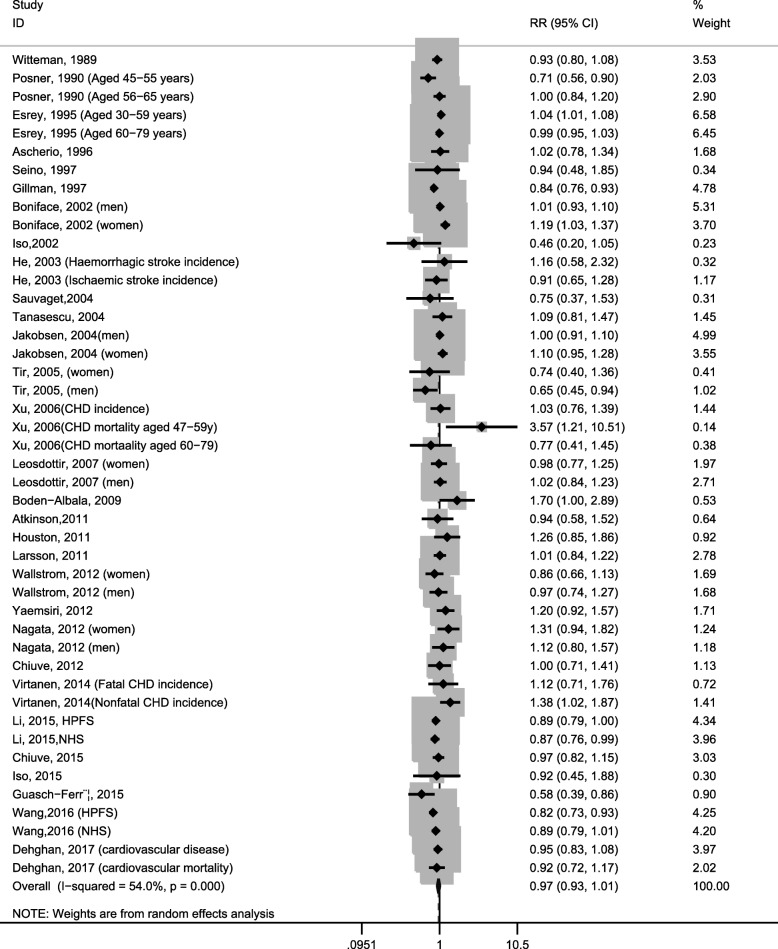
Forest plots of cardiovascular disease for the highest versus lowest categories of dietary total fat intake

Forty-five studies reported the relationship between total dietary fat intake and CVDs risk (CVDs incidence and mortality). Highest versus lowest levels of total dietary fat were not associated with the CVDs risk [0.97(0.93–1.01), *I*^2^ = 54.0%; Fig. 2]. Sensitivity analysis showed that no individual study had an excessive influence on the pooled effect. There was evidence of significant heterogeneity (*I*^2^ = 54.0%), which was further explored in meta-regression. All covariates investigated in the meta-regression provided a poor explanation of the heterogeneity. In addition, the analysis was repeated stratified according to each covariate. The results were consistent with that observed in meta-regression.

### Subgroup analysis

As shown in Table 1, non-significant associations of total dietary fat intake with cardiovascular risk were detected in the subgroup analysis conducted by geographical location (Americas, Europe, or Asia); follow-up duration (< 10 years or ≥ 10 years); And whether the results were adjusted for age, sex, physical activity, energy intake, alcohol, smoking, or body mass index.

**Table 1:** Subgroup analysis of dietary total fat and major fat subclasses and risk of cardiovascular disease

	Dietary total fat			Trans fat			Saturated fatty acid			Monounsaturated fatty acid			Polyunsaturated fatty acid		
	N	HR(95%*CI*)	*I*^*2*^ (%)	N	HR(95%*CI*)	*I*^*2*^ (%)	N	HR(95%CI)	*I*^*2*^ (%)	N	HR(95%*CI*)	*I*^*2*^ (%)	N	HR(95%CI)	*I*^*2*^ (%)
Overall	45	0.97 (0.93–1.01)	54.0	24	1.14 (1.08–1.21)	26.1	56	0.97 (0.93–1.02)	56.8	43	0.97 (0.93–1.01)	50.3	45	0.97 (0.93–1.00)	55.8
Follow-up duration (years)															
< 10	15	0.97 (0.88–1.08)	49.0	10	1.20 (1.10–1.30)	0.0	19	0.97 (0.89–1.05)	48.8	12	0.94 (0.81–1.08)	51.3	15	1.03 (0.94–1.13)	34.0
≥ 10	30	0.97 (0.93–1.01)	57.0	14	1.11 (1.02–1.19)	38.2	37	0.98 (0.93–1.03)	60.9	31	0.98 (0.94–1.02)	50.5	30	0.95 (0.91–0.99)	62.4
Geographical location															
America	23	0.95 (0.90–1.01)	63.2	17	1.13 (1.06–1.21)	24.2	24	1.01 (0.95–1.07)	58.8	20	0.94 (0.88–1.02)	59.7	20	0.93 (0.86–1.01)	60.5
Europe	14	1.00 (0.93–1.08)	50.2	7	1.16 (1.04–1.31)	40.0	21	0.98 (0.91–1.06)	49.5	16	1.01 (0.95–1.06)	42.8	18	1.01 (0.98–1.05)	27.6
Asia	6	1.01 (0.80–1.28)	27.6	0	–	–	9	0.84 (0.73–0.97)	42.3	5	0.90 (0.64–1.27)	33.3	5	1.09 (0.86–1.39)	0.0
Adjust for age															
Yes	32	0.99 (0.95–1.03)	48.4	16	1.13 (1.03–1.23)	35.1	43	0.97 (0.92–1.02)	57.9	30	0.99 (0.95–1.03)	42.7	33	0.99 (0.95–1.02)	48.9
No	13	0.93 (0.86–1.01)	53.0	8	1.16 (1.06–1.28)	9.5	13	0.99 (0.89–1.09)	53.5	13	0.95 (0.85–1.06)	51.2	12	0.91 (0.81–1.03)	43.3
Adjust for sex															
Yes	8	1.01 (0.93–1.09)	61.8	6	1.07 (0.96–1.69)	0.2	21	0.94 (0.87–1.02)	72.6	13	0.96 (0.92–1.01)	63.2	14	1.00 (0.94–1.05)	9.1
No	22	1.00 (0.96–1.04)	43.2	18	1.15 (1.08–1.23)	30.4	35	1.00 (0.94–1.05)	37.4	30	0.95 (0.86–1.06)	42.7	31	0.96 (0.91–1.01)	64.8
Adjust for alcohol															
Yes	12	0.99 (0.92–1.07)	59.5	20	1.13 (1.06–1.19)	24.5	41	0.97 (0.92–1.02)	51.7	29	0.98 (0.93–1.02)	47.5	30	0.96 (0.92–1.01)	64.8
No	33	0.97 (0.92–1.01)	46.6	4	1.30 (1.06–1.61)	23.2	15	0.99 (0.90–1.08)	60.7	14	0.93 (0.85–1.02)	58.2	15	0.99 (0.93–1.01)	18.4
Adjust for energy intake															
Yes	32	0.96 (0.92–1.01)	62.0	18	1.16 (1.08–1.25)	26.3	40	0.96 (0.91–1.02)	64.5	30	0.96 (0.90–1.02)	57.2	31	0.95 (0.89–1.01)	54.0
No	13	1.00 (0.94–1.06)	12.4	6	1.09 (1.00–1.19)	11.1	16	1.01 (0.95–1.08)	6.0	13	1.00 (0.97–1.03)	12.9	14	1.01 (0.98–1.04)	27.2
Adjust for physical activity															
Yes	33	1.00 (0.93–1.08)	56.1	19	1.14 (1.06–1.22)	38.3	43	0.96 (0.92–1.01)	49.8	31	0.94 (0.88–1.00)	42.2	32	0.97 (0.91–1.03)	59.5
No	12	1.02 (0.99–1.04)	22.2	5	1.15 (1.02–1.30)	0.0	13	1.01 (0.91–1.12)	60.6	12	1.02 (0.98–1.06)	41.5	13	1.01 (0.99–1.02)	0.0
Adjust for smoking															
Yes	41	0.97 (0.92–1.01)	57.0	22	1.15 (1.08–1.23)	28.0	52	0.97 (0.92–1.01)	58.3	40	0.97 (0.93–1.01)	51.8	42	0.97 (0.93–1.01)	58.0
No	4	1.01 (0.94–1.08)	0.0	2	1.08 (0.98–1.20)	0.0	4	1.05 (0.95–1.16)	1.0	3	1.08 (0.80–1.46)	33.1	3	0.88 (0.67–1.15)	0.0
Adjust for body mass index															
Yes	35	0.96 (0.91–1.01)	56.4	23	1.14 (1.07–1.21)	29.4	43	0.97 (0.91–1.02)	58.1	36	0.98 (0.94–1.02)	50.3	37	0.96 (0.93–1.01)	59.1
No	10	0.99 (0.93–1.06)	48.1	1	1.14 (0.97–1.35)	–	13	0.99 (0.92–1.07)	55.6	7	0.93 (0.80–1.07)	51.0	8	1.01 (0.87–1.17)	39.8

*Abbreviation*: *HR* hazards ratio

### Publication bias

Egger test showed no evidence of significant publication bias for this meta-analysis with cardiovascular risk and total fat intake (*t* = − 1.01, *P* = 0.319).

### Trans fatty acids intake and risk of CVDs

**Fig. 3:**
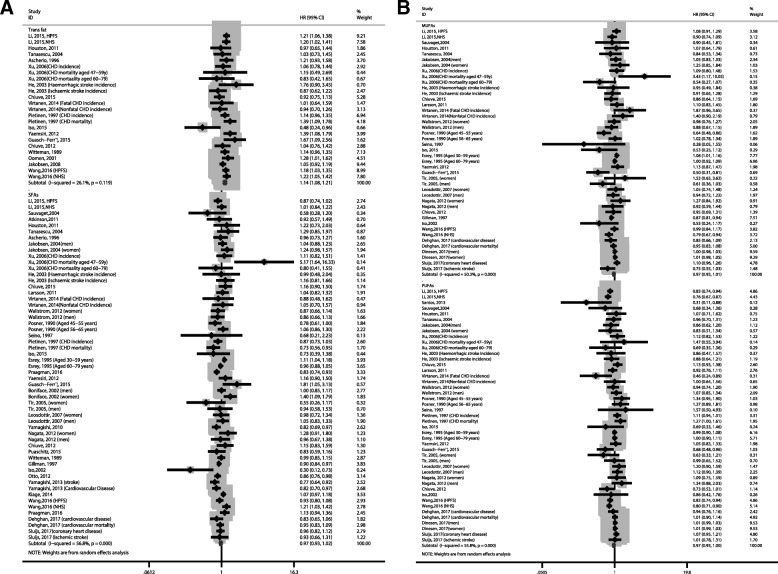
Forest plots of cardiovascular disease for the highest versus lowest categories of dietary trans fatty acids (**a**), saturated fatty acids (**a**), monounsaturated fatty acids (**b**), and polyunsaturated fatty acids intake (**b**)

Twenty-four studies reported the effect of dietary TFA intake on CVDs risk. Highest versus lowest levels of dietary TFA were associated with increased risk of CVDs [1.14(1.08–1.21), *I*^2^ = 26.1%; Fig. 3a]. Sensitivity analysis showed that no individual study had an excessive influence on the pooled effect.

### Subgroup analysis

In subgroup analysis, the positive association between dietary trans fatty acids intake and CVDs risk were observed in most studies except those were not adjusted for smoking and body mass index, and studies adjusted for sex (Table 1).

### Publication bias

Egger test showed no evidence of significant publication bias for this meta-analysis with cardiovascular risk and TFA intake (*t* = − 1.00, *P* = 0.330).

### Saturated fatty acids intake and risk of CVDs

Fifty-six studies reported the association between dietary SFA intake and CVDs risk. Highest versus lowest levels of dietary SFA were not associated with the risk of CVDs [0.97(0.93–1.02), *I*^2^ = 56.8%; Fig. 3a]. Sensitivity analysis showed that no individual study had an excessive influence on the pooled effect. There was evidence of significant heterogeneity (*I*^2^ = 56.8%), which was further explored in meta-regression. All covariates investigated in the meta-regression provided a poor explanation of the heterogeneity. In addition, the analysis was repeated stratified according to each covariate. The results were consistent with that observed in meta-regression.

### Subgroup analysis

As shown in Table 1, a significant inverse association was observed in Asia population [0.84(0.73–0.97), *I*^2^ = 42.3%] but not American and European population. No significant associations were found for subgroups by follow-up duration (< 10 years or ≥ 10 years), or whether the results were adjusted for age, sex, physical activity, energy intake, alcohol, smoking, or body mass index (*P* > 0.05).

### Publication bias

Egger test showed no evidence of significant publication bias for this meta-analysis with cardiovascular risk and saturated fatty acids intake (*t* = − 0.28, *P* = 0.777).

### Monounsaturated fatty acids intake and risk of CVDs

Forty-three studies reported the relationship between dietary MUFA intake and CVDs risk. Highest versus lowest levels of dietary MUFA were not associated with the risk of CVDs risk [0.97(0.93–1.01), *I*^2^ = 50.3%; Fig. 3b]. Sensitivity analysis showed that no individual study had an excessive influence on the pooled effect. There was evidence of significant heterogeneity (*I*^2^ = 50.3%), which was further explored in meta-regression. All covariates investigated in the meta-regression provided a poor explanation of the heterogeneity. In addition, the analysis was repeated stratified according to each covariate. The results were consistent with that observed in meta-regression.

### Subgroup analysis

Non-significant associations of dietary monounsaturated fatty acids intake and cardiovascular risk were detected in the subgroup analysis conducted by geographical location (Americas, Europe, or other), follow-up duration (< 10 years or ≥ 10 years), and whether the results were adjusted for age, sex, physical activity, energy intake, alcohol, smoking, or body mass index (Table 1).

### Publication bias

Egger test showed no evidence of significant publication bias for this meta-analysis with cardiovascular risk and monounsaturated fatty acids intake (*t* = − 1.45, *P* = 0.154).

### Polyunsaturated fatty acids intake and risk of CVDs

Forty-five studies reported the effect of dietary PUFA intake on CVDs risk. Highest versus lowest levels of dietary PUFA were not associated with the risk of cardiovascular disease [0.97(0.93–1.004), *I*^2^ = 55.8%; Fig. 3b]. Sensitivity analysis showed that no individual study had an excessive influence on the pooled effect. There was evidence of significant heterogeneity (*I*^2^ = 55.8%), which was further explored in meta-regression. All covariates investigated in the meta-regression provided a poor explanation of the heterogeneity. In addition, the analysis was repeated stratified according to each covariate. The results were consistent with that observed in meta-regression.

### Subgroup analysis

In subgroup analysis, a significant inverse association was observed in the studies that has been followed up more than 10 years [0.95(0.91–0.99), *I*^2^ = 62.4%] (Table 1). No significant associations were found for the other subgroups analysis (*P* > 0.05).

### Publication bias

Egger test showed no evidence of significant publication bias for this meta-analysis with cardiovascular risk and polyunsaturated fatty acids intake (*t* = − 1.74, *P* = 0.088).

### Dose-response analysis

**Fig. 4:**
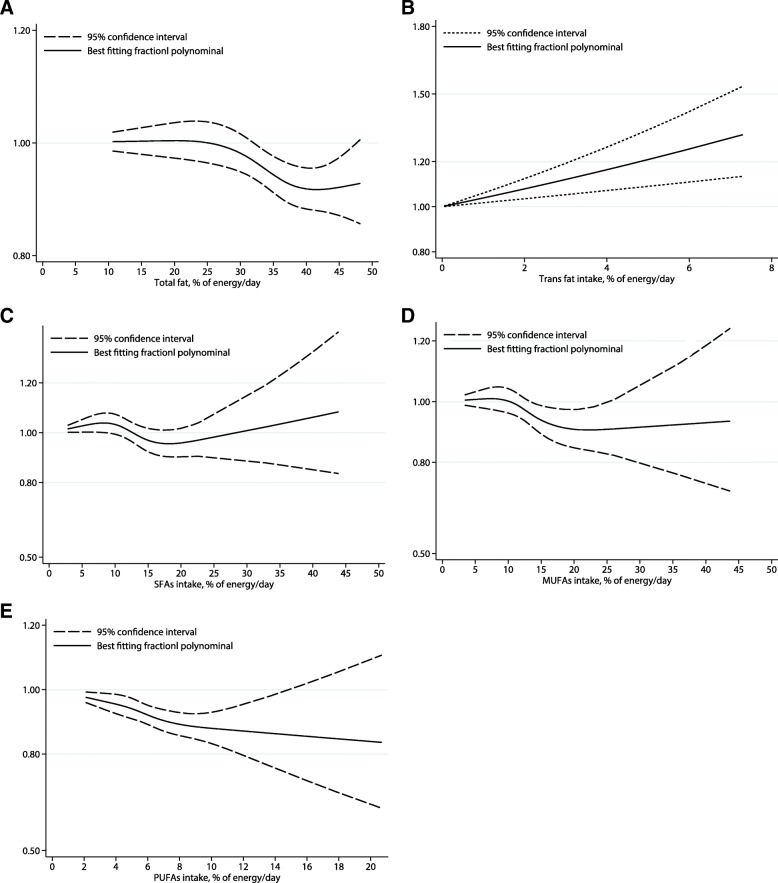
Dose-response analyses of the linear association between dietary total fat (**a**), trans fatty acids (**b**), saturated fatty acids (**c**), monounsaturated fatty acids (**d**), and polyunsaturated fatty acids intake (**e**) and the risk of cardiovascular disease (% energy/day)

**Fig. 5:**
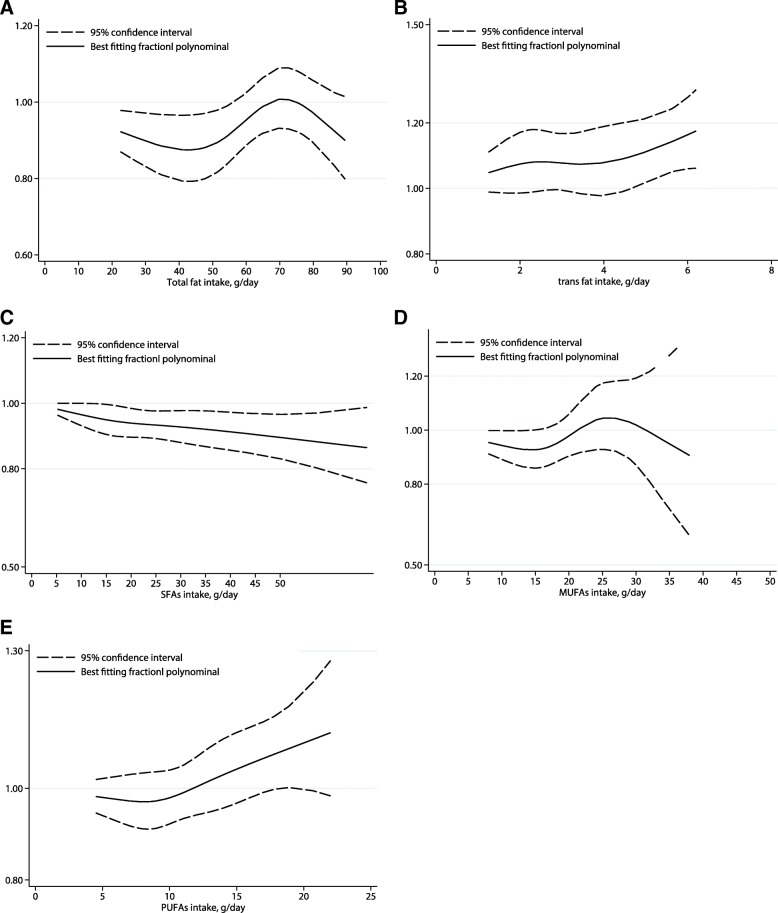
Dose-response analyses of the linear association between dietary total fat (**a**), trans fatty acids (**b**), saturated fatty acids (**c**), monounsaturated fatty acids (**d**), and polyunsaturated fatty acids intake (**e**) and the risk of cardiovascular disease(g/d)

The dose-response analysis suggested non-significant linear relationship between dietary total fat, SFA, MUFA, and PUFA and CVDs risk [0.99(0.97–1.01), *P*_linearity_ = 0.164 and 1.01(0.99–1.02), *P*_linearity_ = 0.848 for an increment of 5% energy and 5 g/day of total dietary fat, respectively; 0.99(0.95–1.04), *P*_linearity_ = 0.474 and 0.98 (0.95–1.00), *P*_linearity_ = 0.526 for an increment of 5% energy and 5 g/day of SFA, respectively; 0.96(0.91–1.02), *P*_linearity_ = 0.471 and 1.00 (0.94–1.06), *P*_linearity_ = 0.256 for an increment of 5% energy and 5 g/day of MUFA, respectively; 0.92(0.84–1.01), *P*_linearity_ = 0.757 for an increment of 5% energy of dietary PUFA intake). However, the risk of CVDs increased 16% [1.16(1.07–1.25), *P*_linearity_ = 0.033], and 4% [1.04(1.01–1.07), *P*_linearity_ = 0.030] for an increment of 2% energy of dietary TFA intake and 5 g/day dietary PUFA intake, respectively (Figs. 4, 5, Additional file 2: Figure S1, Additional file 3: Figure S2, Additional file 4: Figure S3, Additional file 5: Figure S4, and Additional file 6: Figure S5).

## Discussion

This is the first study to investigate the effect of dietary total fat and fatty acid intake on CVDs risk based on dose-response meta-analysis of prospective cohort studies. Results from this meta-analysis did not detect any relationship of dietary total fat, PUFA, MUFA, and SFA intake with CVD risk. However, we found that dietary TFA intake had a dose-response association with CVDs risk. Subgroup analysis suggested inverse associations between PUFAs intake and CVDs risk among studies followed up for more than 10 years.

In contrast to popular accepted viewpoints, we did not detect a positive association between total dietary fat intake and CVDs risk. For decades, it has been believed that fat intake was a risk factor for CVDs, based on the presumption that dietary fat can increase low density lipoprotein (LDL) cholesterol and blood pressure, therefore increase CVDs risk. This viewpoint might be based on selective emphasis on some studies [25, 40, 47], but ignore other studies that do not support these conclusion [15, 36, 51]. Previous meta-analysis also suggested no association between total dietary fat intake and endometrial cancer [63], ulcerative colitis [64], and breast cancer mortality [65], adding new information to reconsider the recommendations of dietary guidelines to reduce fat intake. Even though this study does not support the popular viewpoint that dietary fat intake could increase CVDs risk, it is possible that the role of dietary fat played in the development of CVDs might be confounded by the fat sources. For instance, vegetables and fruits play protective roles in the development of CVDs. However, we could not investigate the different effects of fat from animal, vegetables and fruit separately in this current meta-analysis.

Skeaff et.al. found that dietary TFA intake can increase risk of CHD mortality and incidence [1.32 (1.08–1.61, *P* = 0.006) and 1.25(1.07–1.46) *P* = 0.007), respectively] [66], which is consistent with our meta-analysis. The potential mechanism might be via inflammation process and blood lipids [67]. A recent randomized controlled clinical (RCT) showed that the reduction in TFA intake over 1 year was significantly associated with a reduction in LDL particle number (LDL-P), a novel marker of CVD risk [68]. The meta-analysis of 7 RCTs suggested that increased TFA intake lead to an increase in total and LDL-cholesterol and a decrease in HDL-cholesterol concentrations [69]. Some studies have also demonstrated that increased TFA intake may impair insulin sensitivity [70], increase soluble tumor necrosis factor alpha receptors 1 and 2 (sTNF-R1, sTNF-R2, [71]), and C-reactive protein concentrations [72].

We found no significant association between dietary PUFA or SFA intake and CVDs risk, subgroup analysis suggested that the relative risk of CVDs in the highest compared with the lowest categories of PUFA intake was reduced by 5% in studies that has been followed up for more than 10 years, which suggested that the nonsignificant cardio-protective effect of PUFA might be ascribed to the relatively short follow up duration. The non-significant associations between PUFA and CVD risk detected by a recent research conducted by PURE [18] from five continents (mean follow up time:7.4 years) might be also ascribed to the relative short follow up time. All of these evidences demonstrated that studies with longer follow up time are needed to clarify the effect of dietary PUFA on CVDs risk.

The dose-response analysis suggested a linear relationship between dietary TFA and the risk of CVDs. However, we found no evidence for the linear effects of dietary total fat, SFA, MUFA intake on CVDs risk, supporting the validity of the main analysis results that there is no significant effect of total dietary fat, MUFA, and SFA on the risk of CVDs. What’s surprising, the dose-response analysis found a positive dose-response relationship among studies based on dietary PUFA intake as a g/day but not % of energy/day. Thus, we studied each individual study that based on dietary PUFA intake as a g/day respectively, finding only one study showed a significant association between dietary PUFA intake and CVDs risk [highest vs. lowest RR: 1.27 (1.00–1.61)], and the author suggested that the harmful effect might be ascribed the high content of mercury in local fish, a major source of PUFA. Further study adjusted mercury exposure are needed to clarify the hypothesis.

Potential limitations to this study should also be considered. As common in meta-analysis, publication bias is inevitable. However, the funnel plots and Begg’s test suggested no significant publication bias through. In addition, dietary data from most studies were collected by FFQ, which may introduce measurement errors by the over- or under-reporting of the amounts of food that they usually eaten every day. Thirdly, covariates adjustment could also influence the association between fat and CVDs risk, although we extracted the risk estimates that adjusted the greatest degree of potential confounders which varied among different studies. Forth, we found significant heterogeneity in our study. However, previous studies suggested that when there are many studies with large sample sizes, the *I*^*2*^ test may detect statistically significant but clinically nonsignificant heterogeneity, which may be the case in our study [73]. What’s more, we also explored potential sources of heterogeneity through subgroup and meta-regression analysis, which further proves that our findings are robust.

Our analysis has several strengths. We limited our analysis to prospective cohort studies to minimize the influence of recall and selection biases that are common in case-control studies. Moreover, we conducted a dose-response curve instead of only comparing highest vs. lowest fat consumers. The health effect of diet fat may vary among different fat types. Therefore, it is another strength to combine studies that have investigated the effect of different major dietary fat types on CVDs.

## Conclusions

This current meta-analysis of cohort studies suggested that total fat, SFA, MUFA, and PUFA intake were not associated with the risk of cardiovascular disease. However, we found that higher TFA intake is associated with greater risk of CVDs in a dose-response fashion. Furthermore, the subgroup analysis found a cardio-protective effect of PUFA in studies followed up for more than 10 years. Dietary guidelines taking these findings into consideration might be more credible.

## Additional files

Additional file 1: **Table S1.** Characteristics of included cohort studies reporting CVDs risk and fat intake. (DOCX 82 kb)

Additional file 2: **Figure S1.** Dose-response forest plot of every 5 energy/day (or 5 g/day) increased intake of total dietary fat and the risk of cardiovascular disease. (EPS 2101 kb)

Additional file 3: **Figure S2.** Dose-response forest plot of every 2 energy/day (or 2 g/day) increased intake of dietary tans fatty acids and the risk of cardiovascular disease. (EPS 1709 kb)

Additional file 4: **Figure S3.** Dose-response forest plot of every 5 energy/day (or 5 g/day) increased intake of dietary saturated fatty acids and the risk of cardiovascular disease. (EPS 1904 kb)

Additional file 5: **Figure S4.** Dose-response forest plot of every 5 energy/day (or 5 g/day) increased intake of dietary monounsaturated fatty acids and the risk of cardiovascular disease. (EPS 48 kb)

Additional file 6: **Figure S5.** Dose-response forest plot of every 5 energy/day (or 5 g/day) increased intake of dietary polyunsaturated fatty acids and the risk of cardiovascular disease. (EPS 2084 kb)

## Abbreviations

CVD: Cardiovascular diseases
FFQ: Food frequency questionnaire
HR: Hazard ratio
JACC: Japan Collaborative Cohort Study for Evaluation of Cancer Risk
LDL: Low density lipoprotein
LDL-P: LDL particle number
MedDiets: Mediterranean diets
MUFA: Monounsaturated fatty acids
PREDIMED: REvención con DIeta MEDiterránea study
PUFA: Polyunsaturated fatty acids
PURE: The Prospective Urban Rural Epidemiological
RR: Relative risk
SFA: Saturated fatty acids
sTNF-R1: Soluble tumor necrosis factor alpha receptors 1
sTNF-R2: Soluble tumor necrosis factor alpha receptors 2
TFA: Trans fatty acids
