# Supplementary material for: Dietary total fat, fatty acids intake, and risk of cardiovascular disease: a dose-response meta-analysis of cohort studies
Source: Lipids Health Dis. 2019 Apr 6;18:91. doi: 10.1186/s12944-019-1035-2 (PMC6451787; doi:10.1186/s12944-019-1035-2)
Supplement: Supplementary file 1 — Table S1. Characteristics of included cohort studies reporting CVDs risk and fat intake. (DOCX 82 kb) [file 12944_2019_1035_MOESM1_ESM.docx]

Table S1. Characteristics of included cohort studies reporting CVDs risk and fat intake

| Lead author, year: cohort (country) | Mean age (range), y | Number analyzed (% male) | Outcome (follow-up) | Dietary assessment | Maximum adjusted covariates |
| --- | --- | --- | --- | --- | --- |
| Witteman, 1989: Nurses' Health Study(USA)[1] | NR (34-59) | 60,200 (0) | Hypertension (4) | 61-item FFQ | age, Quetelet's index, alcohol consumption, intakes of calcium, and magnesium |
| Posner, 1990: The Framingham Study (USA)[2] | 55.7 (45-65) | 813(100) | CHD incidence (16) | 24-hour dietary | Dietary variable of interest, energy intake, physical activity, serum cholesterol level, systolic blood pressure, left ventricular hypertrophy, cigarette smoking, glucose intolerance, and Metropolitan relative weight |
| Esrey, 1995: LRCPFS (USA)[3] | NR (30-79) | 4150(57) | CHD mortality (12.4) | 24-hour dietary recall | Age, sex, energy intake, serum lipids, SBP, smoking, BMI, and glucose intolerance. |
| Ascherio, 1996: HPFS (USA)[4] | 40-75 | 43,757(100) | Myocardial infarction incidence (6) | 131 item-FFQ | age, body mass index, smoking habits, alcohol consumption, physical activity, history of hypertension or high blood cholesterol; family history of myocardial infarction before age 60, profession, fibre intake adjusted for energy |
| Pietinen, 1997: ATBC (Finnish)[5] | NR (50-69) | 21930(100) | CHD incidence and mortality (6.1) | 276-item FFQ | Age, smoking, BMI, BP, intakes of energy, alcohol, and fiber, education, and physical activity |
| [Seino](https://www.ncbi.nlm.nih.gov/pubmed/?term=Seino%20F%5BAuthor%5D&cauthor=true&cauthor_uid=9151243), 1997: The Shibata Study (Japan)[6] | NR (40-89) | 2283(42) | Cerebral Infarction incidence (15.5) | 66-item FFQ | Sex, age, diastolic blood pressure and atrial fibrillation |
| Gillman, 1997: Framingham Heart Study (USA)[7] | 55.8±4.6(45-65) | 832 (100) | Stroke incidence (20) | 24-hour dietary recall | Systolic blood pressure, cigarette smoking, glucose intolerance, body mass index, physical activity index, left ventricular hypertrophy, and intake of energy, alcohol, and fruits and vegetables |
| Oomen, 2001: Zuphen Elderly Study (Netherland)[8] | 64–84 | 667 (100) | Coronary heart disease incidence (10) | Cross-check dietary history method | age, body mass index, smoking, and dietary covariates, trans fatty acid intake at baseline |
| Boniface, 2002: The Health and Lifestyle Survey (UK)[9] | NR (40-75) | 2676 (46) | CHD mortality (16) | 30-item FFQ | Age, alcohol consumption, smoking, exercise, and social class |
| Iso, 2002: NA (Japan)[10] | 40-69 | 4755 (47.72) | Intraparenchymal hemorrhage incidence (14.3) | 24-hour dietary recall | age, sex, quartiles of total energy intake and body mass index, hypertension category, diabetes, serum total cholesterol, smoking status, ethanol intake, and (for women) menopausal status |
| He, 2003: HPFS (USA)[11] | NR(40-75) | 43732(100) | Stroke incidence (14) | 131item-FFQ | BMI, physical activity, history of hypertension, smoking status, aspirin use, multivitamin use, alcohol, potassium, fibre, vitamin E, total servings of fruit and vegetables, energy intake, and hypercholesterolaemia at baseline. |
| Sauvaget,2004: AHS (Japan)[12] | 57(35-89) | 3731(39) | Cerebral Infarction mortality (14) | 24-hour dietary survey | Radiation dose, city, BMI, smoking, alcohol, medical history of hypertension and diabetes |
| Tanasescu,2004: NHS (USA)[13] | 30-55 | 5672(0) | Fatal CHD, nonfatal myocardial infarction, and stroke (10.1) | 61 item-FFQ | age, smoking, postmenopausal hormone use, parental history of myocardial infarction before 60 y of age, alcohol intake, moderate/vigorous activities, BMI, total caloric intake, protein intake, fiber intake, multivitamin use, vitamin E supplement use, and medication use. |
| Jakobsen, 2004: Danish MONICA I & III (Denmark)[14] | NR (30-71) | 3686(53) | CHD incidence (16) | 7-day  weighed food record | The percentage of total energy intake, total energy intake, cohort identification, the percentage of energy derived from protein from the other major types of fatty acids, and the other nondietary and dietary coronary heart disease risk factors |
| Leosdottir, 2005: MDC (Sweden)[15] | 45-73 | 27959 (39.36) | Cardiovascular mortality (6.6) | 53-item FFQ | age, alcohol consumption, smoking, social class, marital status, physical activity, BMI and fibre intake |
| Xu, 2006: SHS (USA)[16] | 53.6 (47–79) | 2938(36) | CHD incidence and mortality (7.2) | 24-h diet recall | Percentage of energy, sex, age, study center, diabetes status, BMI, HDL, LDL, TG, smoking, alcohol, hypertension, percentage of energy from protein, and total energy intak |
| Leosdottir, 2007: MDC (Sweden)[17] | NA | 28098 (39.40) | cardiovascular events (8.4) | 53-item FFQ | age, smoking habits, alcohol consumption, socioeconomic status, marital status, physical activity, body mass index, fibre intake, and blood pressure. |
| Jakobsen, 2008: NA (Denmark)[18] | 30-71 | 3686 (50.62) | CHD incidence (18) | 7-day weighed food record | energy-adjusted intake of R-TFA, total energy intake, cohort identification, sex, body mass index, systolic blood pressure, familial history of myocardial infarction, education, leisure-time physical activity, smoking, alcohol intake, percentages of energy intake from protein, monounsaturated fatty acids and polyunsaturated fatty acids, dietary fibre intake, dietary cholesterol intake, weighted intake of foods containing high amounts of industrially produced trans fatty acids, and percentage of energy intake from saturated fatty acids |
| Albala, 2009: NOMAS (USA)[19] | 69.1±10 | 3183 (47) | Ischemic stroke incidence (5.5) | 207-item FFQ | age, race/ethnicity, sex, education, hypertension, diabetes, coronary artery disease, moderate alcohol, smoking, BMI,leisure physical activity, dietary intake of sodium, potassium, fruit/vegetable calcium, fiber, vitamin E, calories |
| Yamagishi, 2010: JACC (Japan)[20] | 55.9 (40–79) | 58453(39) | cardiovascular mortality (14.1) | 33 item FFQ | Age, sex, history of hypertension and diabetes, smoking status, alcohol consumption, BMI, mental stress, walking, sports, educational level, total energy intake, cholesterol, n-3 and n-6 PUFAs, vegetables, and fruit. |
| Atkinson,2011: CaPS (Japan)[21] | 53.3(45-59) | 2710(100) | Stroke incidence (18) | FFQ^*^ | Age, total energy, smoking, adult social class, marital status, alcohol; vitamin C, vegetable and fibre intake; BP, cholesterol, BMI, fasting glucose, diabetes, atrial fibrillation |
| Larsson, 2011: SMC (Sweden)[22] | NR (49–83) | 34670(0) | Stroke incidence (10.4) | 96-item FFQ | Age, smoking status and pack-years of smoking, education, body mass index, total physical activity, history of hypertension, history of diabetes, aspirin use, family history of myocardial infarction, and intakes of alcohol, protein, and dietary fiber,cholesterol |
| Houston, 2011: The Health ABC Study (USA)[23] | 74.5 (70-79) | 1941(45) | Cardiovascular incidence (9) | 108-FFQ | Age, gender, race, education, field center, smoking, alcohol use, physical activity, BMI, total energy, protein, and fiber intake; multivitamin, supplemental vitamin E, statin, and aspirin use, and oral estrogen use (women only); and prevalent diabetes or hypertension, dietary cholesterol |
| Wallstrőm, 2012: MDC (Sweden)[24] | 57.2±7.9 | 8139(100) | Ischemic cardiovascular event (13.5) | 168-item FFQ | Age, method version, total energy intake, season, BMI class, smoking category, education, alcohol category, SBP, antihypertensive treatment, antihyperlipidemic treatment, leisure time physical activity and quintiles of energy-adjusted dietary fiber |
| Chiuve, 2012: NHS (USA)[25] | NR (34–59) | 91981(0) | cardiovascular mortality (30) | 61-item FFQ | Age, total calories, smoking, BMI, family history of MI, menopausal status, hormone therapy, exercise, aspirin use, use of multivitamins and vitamin E supplements, history of diabetes, hypertension, hypercholesterolemia, CHD, and cancer at baseline |
| Otto, 2012: MESA (USA)[26] | 45-84 | 5209 (59.6) | Cardiovascular disease incidence (7) | 120-item FFQ | age, sex, race ethnicity, study center, energy intake, education, alcohol intake, physical activity, BMI, cigarette smoking, dietary supplement use, use of cholesterol-lowering medication, intakes of fruit and vegetables, and energy adjusted intakes of dietary fiber, dietary vitamin E, trans fat, and PUFA |
| Nagata, 2012: The Takayama Study (Japan)[27] | NR (30~) | 28356 (54) | Cardiovascular mortality (16) | 169-item FFQ | Age, non-alcohol energy, height, BMI, physical activity, smoking status, alcohol intake, education, marital status, histories of diabetes and hypertension, protein expressed as percentage of non-alcohol energy, and intakes of fruits, vegetables, and dietary fiber. |
| Yaemsiri, 2012: WHI-OS (Columbia)[28] | 63.5 (50-79) | 87025(0) | Stroke incidence (7.6) | 122-item FFQ | age and race, education, family income, years as a regular smoker, hormone replacement therapy use, total MET hours per wk, alcohol intake, history of coronary heart disease, history of atrial fibrillation, history of diabetes, aspirin use, use of antihypertensive medication, use of cholesterol-lowering medication, BMI, systolic blood pressure, and total energy intake, dietary vitamin E, fruits and vegetable intake, fiber |
| Santos, 2013:NR (Brazil)[29] | 59 ± 10 | 227(46) | Cardiovascular events (4.6) | 3-day weighed diet records | age, gender, duration of diabetes, smoking, compliance with WDR, using hypolipidemic agents, and the presence of hypertension and diabetic nephropathy |
| Yamagishi, 2013: JPHC (Japan)[30] | 56.7 | 81931(46.48) | Stroke and other Cardiovascular Disease incidence (11.1) | 138-item FFQ | age; sex; energy intake; cohort; cigarette smoking status; alcohol intake; body mass index; sports at leisure time; walking and standing time; perceived mental stress; energy-adjusted dietary intakes of carbohydrate, protein, cholesterol, vegetables, fruit, and calcium |
| Virtanen, 2014: KIHD (Finland)[31] | 52.5 (42-60) | 1981(100) | CHD incidence (21.4) | Instructed food recording of four consecutive days | Age, examination year and energy intake, BMI, diabetes mellitus, hypertension, family history of CHD, pack-years of smoking, education, leisure-time physical activity, intakes of alcohol and fiber, percentage of energy from protein; percentage of energy from remaining fatty acids (saturated fatty acids, trans fatty acids, monounsaturated fatty acids, and polyunsaturated fatty acids) were further adjusted for quintiles of saturated fatty acids, trans fatty acids, monounsaturated fatty acids, and polyunsaturated fatty acids |
| Kiage, 2014: REGARDS (USA)[32] | 64.6±9.1 | 13686 (30.00) | Stroke incidence (7) | Self-administered Block 1998 food-frequency questionnaire | sex, age, and smoking status, race, age, region, alcohol use, education, waist circumference, level of physical activity, diabetes, ischemic heart disease, hypertension, baseline stroke, heart failure, kidney failure, statin use, regular aspirin use, total energy and energy-adjusted saturated fat, monounsaturated fat, polyunsaturated fat, and protein |
| Li, 2015: HPFS and NHS (USA)[33] | HPFS: 40-75;  NHS: 30-55 | HPFS:41908(100)  NHS: 84628(0) | CHD mortality (HPFS:24; NHS:30) | HPFS: 131item-FFQ; NHS: 61-item-FFQ | Total energy intake, the energy contribution from protein, cholesterol intake, alcohol intake, smoking status, BMI, physical activity, use of vitamins and aspirin, family history of MI and diabetes, and presence of baseline hypercholesterolemia and hypertension |
| Chiuve, 2015: WHS (USA)[34] | 45- | 33665(0) | Sustained Atrial Fibrillation (19.2) | 131-item FFQ | age, protein (percentage of energy), total calories, smoking, BMI, height, alcohol, exercise, education, race, randomization group (b-carotene, vitamin E, and aspirin), systolic blood pressure, and diagnosis of hypertension, high cholesterol, and diabetes, all at baseline, diagnosis of hypertension, high cholesterol, diabetes, cardiovascular disease, or congestive heart failure during follow-up,SFAs, MUFAs, total PUFAs, and trans fatty acid |
| Iso 2015: NHS (USA)[35] | 34-59 | 85764(0) | Intraparenchymal Hemorrhage incidence (14) | 61-item FFQ | age, sex, intervention group, yearly updated total energy intake, alcohol intake, updated quintiles of fiber, protein intake, and dietary cholesterol for the total fat analysis, BMI, smoking status, educational level, leisure-time physical activity, baseline diabetes, hypertension, hypercholesterolemia, family history of coronary heart disease, use of antihypertensive medication, use of oral antidiabetic agents, and use of lipid-lowering drugs |
| Guasch-Ferré, 2015: PREDIMED (Spain)[36] | 67 (55-80) | 7038(43) | Cardiovascular incidence and mortality (6) | 137-item FFQ | Age, sex, intervention group, intake of energy, alcohol, fiber, protein, dietary cholesterol for the total fat analysis, BMI, smoking status, educational level, leisure-time physical activity, baseline diabetes, hypertension, hypercholesterolemia, family history of CHD, use of antihypertensive medication, oral antidiabetic agents, and lipid-lowering drugs |
| Puaschitz, 2015: WENBIT (Norway)[37] | 61.7 (NR) | 2412(81) | CHD incidence and mortality (4.8) | 169-item FFQ | acute coronary syndrome, age, diabetes mellitus, hypertension, left ventricular ejection fraction, sex, current smoker, and current use of statins |
| Praagman, 2016: EPIC-NL (Dutch)[38] | 49.2 (49–70) | 35597(25) | Ischemic heart disease incidence (12) | 79-item FFQ | Intakes of total carbohydrates, cis MUFAs, PUFAs, trans fat, animal protein, vegetable protein, and total energy (excluding energy from alcohol intake), age, sex, BMI, waist circumference, educational level, physical activity level, smoking status, alcohol intake,energy-adjusted intakes of cholesterol, fiber, and vitamin C. |
| Wang,2016: HPFA and NHS (USA)[39] | HPFS: 40-75;  NHS: 30-55 | HPFS: 42,884 (100)  NHS: 83,349 (0) | Cardiovascular disease mortality (HPFS:26; NHS:32) | HPFS: 131item-FFQ; NHS: 61-itemFFQ | age, Caucasian, marital status, body-mass index, physical activity, smoking status, alcohol consumption, multivitamin use, vitamin E supplementation use, current aspirin use, family history of myocardial infarction, family history of diabetes, family history of cancer, history of hypertension, history of hypercholesterolemia, intakes of total energy, dietary cholesterol and percentage of energy intake from dietary protein, and menopausal status and hormone use in women |
| Praagman, 2016: the Rotterdam Study (Dutch)[40] | 66.2 ±7.4 | 4722 (37.99) | Coronary heart disease incidence (16.3) | 170-item FFQ | age sex, total energy intake, BMI, waist circumference, education level, income level, physical activity, smoking status, and alcohol intake, intakes of trans-fat, animal protein, vegetable protein, energy-adjusted intakes of vitamin C, fiber, and cholesterol, and for the sum of all other SFA. |
| Dehghan, 2017: PURE (18 countries from five continents)[41] | 35-70 | 135,335 (41.7) | Major cardiovascular disease and cardiovascular mortality (7.4) | FFQ* | age, sex, education, waist-to-hip ratio, smoking, physical activity, diabetes, urban or rural location, and energy intake. Centre was also included as a random effect and frailty models |
| Dinesen, 2017: Danish cohort study Diet, Cancer and Health (Denmark)[42] | 50-64 | 27,178(47.64) | Atrial Fibrillation (17) | 192 item-FFQ | age, body mass index, waist circumference, smoking, alcohol, years in school, hypertension, hypercholesterolemia, diabetes mellitus, angina pectoris, previous myocardial infarction, heart failure, renal disease |
| Sluijs, 2017: EPIC-NL (Dutch)[43] | 20-70 | 36520(25.38) | CHD and Ischemic stroke incidence (15) | 178 item-FFQ | age, sex, smoking, physical activity index, BMI, education, systolic blood pressure, hypertension, diabetes, dietary intakes of energy, cholesterol, carbohydrates and alcohol |

*: the FFQ items were not shown.

Abbreviation: AHS: Adult Health Study; BMI: Body Mass Index; CaPS: Caerphilly Prospective Study; FFQ: Food Frequency Questionnaire; BP: Blood Pressure; MONICA: Monitoring of Trends and Determinants in Cardiovascular Disease; NR: Not Reported; CHD: Coronary Heart Disease; WDR: Weighed Diet Records; EPIC-NL: European Prospective Investigation into Cancer and Nutrition–Netherlands; PUFAs: Polyunsaturated fatty acid; MUFAs: Monounsaturated fatty acid; HPFS: Health Professionals Follow-up Study; MI: Miocardial Infarction; JACC: The Japan Collaborative Cohort Study for Evaluation of Cancer Risk; KIHD: The Kuopio Ischemic Heart Disease Risk Factor Study; LRCPFS: Lipid Research Clinics Prevalence Follow-up Study; SBP: Systolic Blood Pressure; MDC: Malmő Diet and Cancer; NHS: Nurses’ Health Study; PREDIMED: the PREvención con DIeta MEDiterránea Study; SHS: The Strong Heart Study; HDL: High density lipoprotein cholesterol; LDL: Low density lipoprotein cholesterol; TG: Triacylglycerol; SMC: Swedish Mammography Cohort; ATBC: The Alpha-Tocopherol, Beta-Carotene Cancer Prevention Study; The Health ABC Study: The Health, Aging and Body Composition Study; WENBIT: The Western Norway B-Vitamin Intervention Trial; WHI-OS: Women’s Health Initiative Observational Study; WHS: Women’ s Health Study

1. Witteman JC, Willett WC, Stampfer MJ, Colditz GA, Sacks FM, Speizer FE, Rosner B, Hennekens CH: **A prospective study of nutritional factors and hypertension among US women**. *Circulation* 1989, **80**(5):1320-1327.

2. Posner BM, Cobb JL, Belanger AJ, Cupples LA, D'Agostino RB, Stokes J, 3rd: **Dietary lipid predictors of coronary heart disease in men. The Framingham Study**. *Arch Intern Med* 1991, **151**(6):1181-1187.

3. Esrey KL, Joseph L, Grover SA: **Relationship between dietary intake and coronary heart disease mortality: lipid research clinics prevalence follow-up study**. *J Clin Epidemiol* 1996, **49**(2):211-216.

4. Ascherio A, Rimm EB, Giovannucci EL, Spiegelman D, Stampfer M, Willett WC: **Dietary fat and risk of coronary heart disease in men: cohort follow up study in the United States**. *BMJ (Clinical research ed)* 1996, **313**(7049):84-90.

5. Pietinen P, Ascherio A, Korhonen P, Hartman AM, Willett WC, Albanes D, Virtamo J: **Intake of fatty acids and risk of coronary heart disease in a cohort of Finnish men. The Alpha-Tocopherol, Beta-Carotene Cancer Prevention Study**. *American journal of epidemiology* 1997, **145**(10):876-887.

6. Seino F, Date C, Nakayama T, Yoshiike N, Yokoyama T, Yamaguchi M, Tanaka H: **Dietary lipids and incidence of cerebral infarction in a Japanese rural community**. *J Nutr Sci Vitaminol (Tokyo)* 1997, **43**(1):83-99.

7. Gillman MW, Cupples LA, Millen BE, Ellison RC, Wolf PA: **Inverse association of dietary fat with development of ischemic stroke in men**. *Jama* 1997, **278**(24):2145-2150.

8. Oomen CM, Ocké MC, Feskens EJM, Erp-Baart M-AJv, Kok FJ, Kromhout D: **Association between trans fatty acid intake and 10-year risk of coronary heart disease in the Zutphen Elderly Study: a prospective population-based study**. *The Lancet* 2001, **357**(9258):746-751.

9. Boniface DR, Tefft ME: **Dietary fats and 16-year coronary heart disease mortality in a cohort of men and women in Great Britain**. *European journal of clinical nutrition* 2002, **56**(8):786-792.

10. Iso H: **Fat and Protein Intakes and Risk of Intraparenchymal Hemorrhage among Middle-aged Japanese**. *American journal of epidemiology* 2003, **157**(1):32-39.

11. He K, Merchant A, Rimm EB, Rosner BA, Stampfer MJ, Willett WC, Ascherio A: **Dietary fat intake and risk of stroke in male US healthcare professionals: 14 year prospective cohort study**. *BMJ (Clinical research ed)* 2003, **327**(7418):777-782.

12. Sauvaget C, Nagano J, Hayashi M, Yamada M: **Animal protein, animal fat, and cholesterol intakes and risk of cerebral infarction mortality in the adult health study**. *Stroke* 2004, **35**(7):1531-1537.

13. Tanasescu M, Cho E, Manson JE, Hu FB: **Dietary fat and cholesterol and the risk of cardiovascular disease among women with type 2 diabetes**. *The American journal of clinical nutrition* 2004, **79**(6):999-1005.

14. Jakobsen MU, Overvad K, Dyerberg J, Schroll M, Heitmann BL: **Dietary fat and risk of coronary heart disease: possible effect modification by gender and age**. *American journal of epidemiology* 2004, **160**(2):141-149.

15. Leosdottir M, Nilsson PM, Nilsson JA, Mansson H, Berglund G: **Dietary fat intake and early mortality patterns--data from The Malmo Diet and Cancer Study**. *J Intern Med* 2005, **258**(2):153-165.

16. Xu J, Eilat-Adar S, Loria C, Goldbourt U, Howard BV, Fabsitz RR, Zephier EM, Mattil C, Lee ET: **Dietary fat intake and risk of coronary heart disease: the Strong Heart Study**. *The American journal of clinical nutrition* 2006, **84**(4):894-902.

17. Leosdottir M, Nilsson PM, Nilsson JA, Berglund G: **Cardiovascular event risk in relation to dietary fat intake in middle-aged individuals: data from The Malmo Diet and Cancer Study**. *European journal of cardiovascular prevention and rehabilitation : official journal of the European Society of Cardiology, Working Groups on Epidemiology & Prevention and Cardiac Rehabilitation and Exercise Physiology* 2007, **14**(5):701-706.

18. Jakobsen MU, Overvad K, Dyerberg J, Heitmann BL: **Intake of ruminant trans fatty acids and risk of coronary heart disease**. *International journal of epidemiology* 2008, **37**(1):173-182.

19. Boden-Albala B, Elkind MS, White H, Szumski A, Paik MC, Sacco RL: **Dietary total fat intake and ischemic stroke risk: the Northern Manhattan Study**. *Neuroepidemiology* 2009, **32**(4):296-301.

20. Yamagishi K, Iso H, Yatsuya H, Tanabe N, Date C, Kikuchi S, Yamamoto A, Inaba Y, Tamakoshi A: **Dietary intake of saturated fatty acids and mortality from cardiovascular disease in Japanese: the Japan Collaborative Cohort Study for Evaluation of Cancer Risk (JACC) Study**. *The American journal of clinical nutrition* 2010, **92**(4):759-765.

21. Atkinson C, Whitley E, Ness A, Baker I: **Associations between types of dietary fat and fish intake and risk of stroke in the Caerphilly Prospective Study (CaPS)**. *Public Health* 2011, **125**(6):345-348.

22. Larsson SC, Virtamo J, Wolk A: **Dietary fats and dietary cholesterol and risk of stroke in women**. *Atherosclerosis* 2012, **221**(1):282-286.

23. Houston DK, Ding J, Lee JS, Garcia M, Kanaya AM, Tylavsky FA, Newman AB, Visser M, Kritchevsky SB: **Dietary fat and cholesterol and risk of cardiovascular disease in older adults: the Health ABC Study**. *Nutrition, metabolism, and cardiovascular diseases : NMCD* 2011, **21**(6):430-437.

24. Wallstrom P, Sonestedt E, Hlebowicz J, Ericson U, Drake I, Persson M, Gullberg B, Hedblad B, Wirfalt E: **Dietary fiber and saturated fat intake associations with cardiovascular disease differ by sex in the Malmo Diet and Cancer Cohort: a prospective study**. *PLoS One* 2012, **7**(2):e31637.

25. Chiuve SE, Rimm EB, Sandhu RK, Bernstein AM, Rexrode KM, Manson JE, Willett WC, Albert CM: **Dietary fat quality and risk of sudden cardiac death in women**. *The American journal of clinical nutrition* 2012, **96**(3):498-507.

26. de Oliveira Otto MC, Mozaffarian D, Kromhout D, Bertoni AG, Sibley CT, Jacobs DR, Jr., Nettleton JA: **Dietary intake of saturated fat by food source and incident cardiovascular disease: the Multi-Ethnic Study of Atherosclerosis**. *The American journal of clinical nutrition* 2012, **96**(2):397-404.

27. Nagata C, Nakamura K, Wada K, Oba S, Tsuji M, Tamai Y, Kawachi T: **Total fat intake is associated with decreased mortality in Japanese men but not in women**. *The Journal of nutrition* 2012, **142**(9):1713-1719.

28. Yaemsiri S, Sen S, Tinker L, Rosamond W, Wassertheil-Smoller S, He K: **Trans fat, aspirin, and ischemic stroke in postmenopausal women**. *Ann Neurol* 2012, **72**(5):704-715.

29. dos Santos AL, Weiss T, Duarte CK, Gross JL, de Azevedo MJ, Zelmanovitz T: **Dietary fat composition and cardiac events in patients with type 2 diabetes**. *Atherosclerosis* 2014, **236**(1):31-38.

30. Yamagishi K, Iso H, Kokubo Y, Saito I, Yatsuya H, Ishihara J, Inoue M, Tsugane S: **Dietary intake of saturated fatty acids and incident stroke and coronary heart disease in Japanese communities: the JPHC Study**. *Eur Heart J* 2013, **34**(16):1225-1232.

31. Virtanen JK, Mursu J, Tuomainen TP, Voutilainen S: **Dietary fatty acids and risk of coronary heart disease in men: the Kuopio Ischemic Heart Disease Risk Factor Study**. *Arteriosclerosis, thrombosis, and vascular biology* 2014, **34**(12):2679-2687.

32. Kiage JN, Merrill PD, Judd SE, He K, Lipworth L, Cushman M, Howard VJ, Kabagambe EK: **Intake of trans fat and incidence of stroke in the REasons for Geographic And Racial Differences in Stroke (REGARDS) cohort**. *The American journal of clinical nutrition* 2014, **99**(5):1071-1076.

33. Li Y, Hruby A, Bernstein AM, Ley SH, Wang DD, Chiuve SE, Sampson L, Rexrode KM, Rimm EB, Willett WC *et al*: **Saturated Fats Compared With Unsaturated Fats and Sources of Carbohydrates in Relation to Risk of Coronary Heart Disease: A Prospective Cohort Study**. *Journal of the American College of Cardiology* 2015, **66**(14):1538-1548.

34. Chiuve SE, Sandhu RK, Moorthy MV, Glynn RJ, Albert CM: **Dietary Fat Intake Is Differentially Associated with Risk of Paroxysmal Compared with Sustained Atrial Fibrillation in Women**. *The Journal of nutrition* 2015, **145**(9):2092-2101.

35. Iso H, Stampfer MJ, Manson JE, Rexrode K, Hu F, Hennekens CH, Colditz GA, Speizer FE, Willett WC: **Prospective study of fat and protein intake and risk of intraparenchymal hemorrhage in women**. *Circulation* 2001, **103**(6):856-863.

36. Guasch-Ferre M, Babio N, Martinez-Gonzalez MA, Corella D, Ros E, Martin-Pelaez S, Estruch R, Aros F, Gomez-Gracia E, Fiol M *et al*: **Dietary fat intake and risk of cardiovascular disease and all-cause mortality in a population at high risk of cardiovascular disease**. 2015, **102**(6):1563-1573.

37. Puaschitz NG, Strand E, Norekval TM, Dierkes J, Dahl L, Svingen GF, Assmus J, Schartum-Hansen H, Oyen J, Pedersen EK *et al*: **Dietary intake of saturated fat is not associated with risk of coronary events or mortality in patients with established coronary artery disease**. *The Journal of nutrition* 2015, **145**(2):299-305.

38. Praagman J, Beulens JW, Alssema M, Zock PL, Wanders AJ, Sluijs I, van der Schouw YT: **The association between dietary saturated fatty acids and ischemic heart disease depends on the type and source of fatty acid in the European Prospective Investigation into Cancer and Nutrition-Netherlands cohort**. *The American journal of clinical nutrition* 2016, **103**(2):356-365.

39. Wang DD, Li Y, Chiuve SE, Stampfer MJ, Manson JE, Rimm EB, Willett WC, Hu FB: **Association of Specific Dietary Fats With Total and Cause-Specific Mortality**. *JAMA Intern Med* 2016, **176**(8):1134-1145.

40. Praagman J, de Jonge EA, Kiefte-de Jong JC, Beulens JW, Sluijs I, Schoufour JD, Hofman A, van der Schouw YT, Franco OH: **Dietary Saturated Fatty Acids and Coronary Heart Disease Risk in a Dutch Middle-Aged and Elderly Population**. *Arteriosclerosis, thrombosis, and vascular biology* 2016, **36**(9):2011-2018.

41. Dehghan M, Mente A, Zhang X, Swaminathan S, Li W, Mohan V, Iqbal R, Kumar R, Wentzel-Viljoen E, Rosengren A *et al*: **Associations of fats and carbohydrate intake with cardiovascular disease and mortality in 18 countries from five continents (PURE): a prospective cohort study**. *Lancet (London, England)* 2017, **390**(10107):2050-2062.

42. Dinesen PT, Joensen AM, Rix TA, Tjonneland A, Schmidt EB, Lundbye-Christensen S, Overvad K: **Effect of Dietary Intake of Saturated Fatty Acids on the Development of Atrial Fibrillation and the Effect of Replacement of Saturated With Monounsaturated and Polyunsaturated Fatty Acids**. *The American journal of cardiology* 2017, **120**(7):1129-1132.

43. Sluijs I, Praagman J, Boer JMA, Verschuren WMM, van der Schouw YT: **Fluidity of the dietary fatty acid profile and risk of coronary heart disease and ischemic stroke: Results from the EPIC-Netherlands cohort study**. *Nutrition, metabolism, and cardiovascular diseases : NMCD* 2017, **27**(9):799-805.
